# VisR Ultrasound Improves Diagnosis of Breast Cancer by the Elastogram-to-B-Mode Ratio in a Blinded Reader Study

**DOI:** 10.1109/ojuffc.2025.3560585

**Published:** 2025-04-14

**Authors:** ANNA V. PHILLIPS, CHERIE M. KUZMIAK, GABRIELA TORRES, CATERINA M. GALLIPPI

**Affiliations:** 1 Lampe Joint Department of Biomedical Engineering, University of North Carolina at Chapel Hill, North Carolina State University, Chapel Hill, NC 27514 USA; 2 Department of Radiology, University of North Carolina at Chapel Hill, Chapel Hill, NC 27514 USA; 3 Siemens Healthineers, Issaquah, WA 98029 USA

**Keywords:** Viscoelastic response, VisR, acoustic radiation force, ARFI, breast cancer diagnosis, E/B ratio, elastography, non-invasive imaging, malignant, benign

## Abstract

Ultrasound elastography is increasingly being used alongside mammography for breast cancer diagnosis, particularly in women with radiographically dense breasts. The elastogram-to-B-Mode ratio (E/B), which compares lesion sizes in B-Mode and stiffness images, has been shown to differentiate malignant (E/B >1) from benign (E/B <1) masses. However, the diagnostic utility of E/B has not yet been evaluated using viscosity images, despite viscosity being an emerging biomarker for malignancy. Furthermore, neglecting viscosity can confound elasticity estimation in viscoelastic media like breast tissue. To address this limitation, we used Viscoelastic Response (VisR) ultrasound to assess the diagnostic value of E/B derived from both relative stiffness (RE) and relative viscosity (RV) images. In a blinded reader study, E/B values from viscosity images exhibited similar trends (>1 in malignant lesions and <1 in benign lesions) to those observed in stiffness images. Additionally, E/B values calculated from RE alone, RV alone, or a combination of RE and RV achieved 4–16% higher AUCs for discriminating malignant lesions compared to E/B derived solely from Acoustic Radiation Force Impulse (ARFI) peak displacement. These results suggest that incorporating VisR-derived viscosity and elasticity metrics into E/B calculations could significantly improve diagnostic accuracy for breast cancer detection.

## I. INTRODUCTION

Breast cancer continues to impose a significant burden on global healthcare, with around 2.3 million new cases diagnosed and 685,000 deaths every year. The number of breast cancer cases is expected to increase by over 40% in the next two decades due to population increase, demographic trends, and increased screening [1]. Fortunately, early detection greatly increases patient survival [2]. Because of this, screening guidelines are frequently revised to balance detecting malignant masses as early as possible while decreasing false positives. Although false positives are less deadly than false negatives, they are still potentially costly and stressful to patients when they result in unnecessary imaging and biopsy. Recent research has shown that by age 40, regular screenings are beneficial and improve the mortality rate compared to the previous guidelines of 45–50 [3]. It is imperative to consider this growing demographic of younger women in the breast cancer screening population when designing imaging protocols.

A related emerging challenge in breast cancer screening and diagnosis is radiographically dense breasts, which are more prevalent in younger women but overall affect 47% of the screening population. Dense, fibrous breast tissue is attenuating to x-rays and results in decreased sensitivity and specificity in detecting lesions through mammography. In these patients, other imaging modalities such as ultrasound and MRI can supplement mammography and improve the accuracy of diagnosing malignant lesions [4]. Ultrasound increases the rate of correct cancer diagnosis in dense breasts compared to mammography alone or mammography with AI detection tools [5]. Ultrasound elastography, which can measure tissue stiffness, further improves the accuracy of diagnosis of malignant lesions in dense breasts. Ultrasound elastography-supplemented mammographic screening had higher specificity, accuracy, and AUC than MRI-supplemented screening in a multi-center trial [6]. In addition, US is widely available, does not require venous access, and is less expensive than MRI.

Ultrasound-derived stiffness is a promising biomarker for breast cancer [7], [8]. However, the most common ultra-sound elastography techniques have drawbacks. Shear wave elastography (SWE) provides quantitative stiffness measurements but requires lateral spatial averaging over millimeters, which can obfuscate small heterogeneous structures. This is a problem for breast cancer diagnosis, as heterogeneities within the tumor or changes at the tumor margin are predictive of malignancy [9]. Strain elastography (SE) qualitatively predicts tissue stiffness based on its displacement after a mechanical stress and does not require the spatial averaging of SWE. It requires either physiological motion or manually applied compression, which can introduce operator dependence. In addition, SE and many SWE techniques ignore tissue viscosity, which is also a biomarker for tumor malignancy [10], [11]. Furthermore, high viscosity can confound stiffness estimates when it is not accounted for in the material model [12].

Our group has developed viscoelastic response (VisR) ultrasound, which separately estimates stiffness (“relative elasticity” or “RE”) and viscosity (“relative viscosity” or “RV”) based on a mass-spring-damper model of tissue displacement [13]. Compared to techniques using manually or physiologically induced tissue displacement, VisR reduces operator dependence by using automated acoustic radiation force impulse (ARFI) pushes. Other advantages of VisR include on-axis estimation of stiffness and viscosity and force delivery directly to the targeted location, which both reduce spatial averaging. The RE and RV parameters are relative to the acoustic radiation force applied to the tissue, which means that RE and RV values should not be compared across images unless forcing conditions are the same. Thankfully, other diagnostically relevant information can be obtained from RE and RV images without the need for equal forcing conditions.

One of the most common elastography-based diagnostic metrics for breast cancer is the elastogram-to-B-Mode ratio (E/B), the ratio of lesion diameter in a stiffness image to lesion diameter in a B-Mode image. In malignant lesions, E/B tends to be >1, whereas in benign lesions it tends to be <1 [14]. Studies have reported high sensitivity and specificity in using E/B to detect malignancy, but results in clinical practice are variable due to differences in technique, interpretation, and ultrasound systems used [15]. Some methods measure lesion diameters in the largest dimension or in a particular orientation like horizontal or vertical [14], [16], [17], [18], [19]. Other methods require that the lesion be measured in the same position in B-Mode and elastogram, but the position is not important [14], [20]. Several studies do not explicitly describe their measurement methods [7], [21], [22]. Clinical implementation of E/B would benefit from clear and objective criteria for lesion measurement as well as more blinded studies to assess reader variability.

The reasons behind E/B separating benign and malignant tumors are not fully understood, but it may be due to changes in mechanical properties in the tumor margin. Malignant lesion boundaries become stiffer due to the desmoplastic response and more viscous due to vascularization and edema [9]. Therefore, it may also be beneficial to evaluate E/B with viscosity images in addition to stiffness images. A preliminary and unblinded analysis from our group found that E/B derived from both VisR RE and RV were significantly different between malignant and benign breast lesions [23].

In this work, we assess the diagnostic benefit of using VisR stiffness and viscosity images to calculate E/B, incorporating two important biomarkers for breast cancer into a quantitative metric. We designed a novel blinded reader study to assess the effectiveness of VisR-derived E/B in comparison to E/B derived from a displacement-based stiffness estimate which does not account for viscosity, ARFI peak displacement (PD). The study design improved upon our previous work by using multiple readers, blinding the readers to information about malignancy, and using objective criteria for lesion measurement. Furthermore, we compared the readers’ individual judgements of lesion measurement criteria with an automated, algorithmic approach to better understand the impact of reader variability. We hypothesize that, in a blinded reader study, E/B derived from VisR RE and RV performs better in a binary classifier model for malignant/benign breast lesion differentiation than E/B derived from ARFI PD.

## II. METHODOLOGY

### A. CLINICAL IMAGING

Thirty-four women with suspicious breast masses scored as BIRADS 4 or 5 by a radiologist were imaged at our institution under IRB approval (IRB #17–0949). Informed consent was obtained for all participants. A double-push VisR imaging sequence was implemented using a Siemens S3000 scanner and 9L4 probe (Siemens Healthineers, Ultrasound Division, Issaquah, WA, USA). The VisR sequence consisted of two reference pulses, two ARFI excitations separated by 8 tracking pulses, and 43 additional tracking pulses. ARFI excitations were 300 cycles at 4.21 MHz, and tracking pulses were 2 cycles at 6.15 MHz with a pulse repetition frequency of 11.5 KHz. The ARFI excitations used F/1.5 focal configuration. Reference and tracking pulses used F/1.5 focal configuration on transmit and F/0.75 on receive. For each data acquisition, the VisR sequence was repeated in 40 evenly spaced lateral positions across a 2 cm field of view to acquire a 2D image.

Imaging focal depth was adjusted to match the location of each patient’s suspicious mass. B-Mode and VisR acquisitions of each lesion were taken at four 30° increments, with the transducer rotated about the direction normal to the skin surface. The transducer was positioned manually by the sonographer with real-time positioning guidance from a gyroscope. A total of 136 *in vivo* lesion images were obtained; 88 were benign and 48 were malignant. Imaging took place prior to the clinically indicated biopsy. Needle core biopsy pathology was used to confirm malignancy or benignity.

### B. DATA PROCESSING

Normalized cross-correlation was used to estimate tissue axial displacement profiles from the RF data. Maximum value of the displacement profiles over time was taken to obtain peak displacement (PD). The displacement data was fit to the 1-D mass-spring-damper VisR model to obtain the parameters relative elasticity (RE) and relative viscosity (RV) [13]. Depth and elasticity corrections were also applied to RE and RV as in previous work [24]. Finally, the B-Mode images were shown to an experienced radiologist, blinded to information about malignancy, who delineated the lesion boundaries using a custom MATLAB GUI, The MathWorks, Inc., Natick, MA, USA.

### C. READER STUDY

**FIGURE 1.**
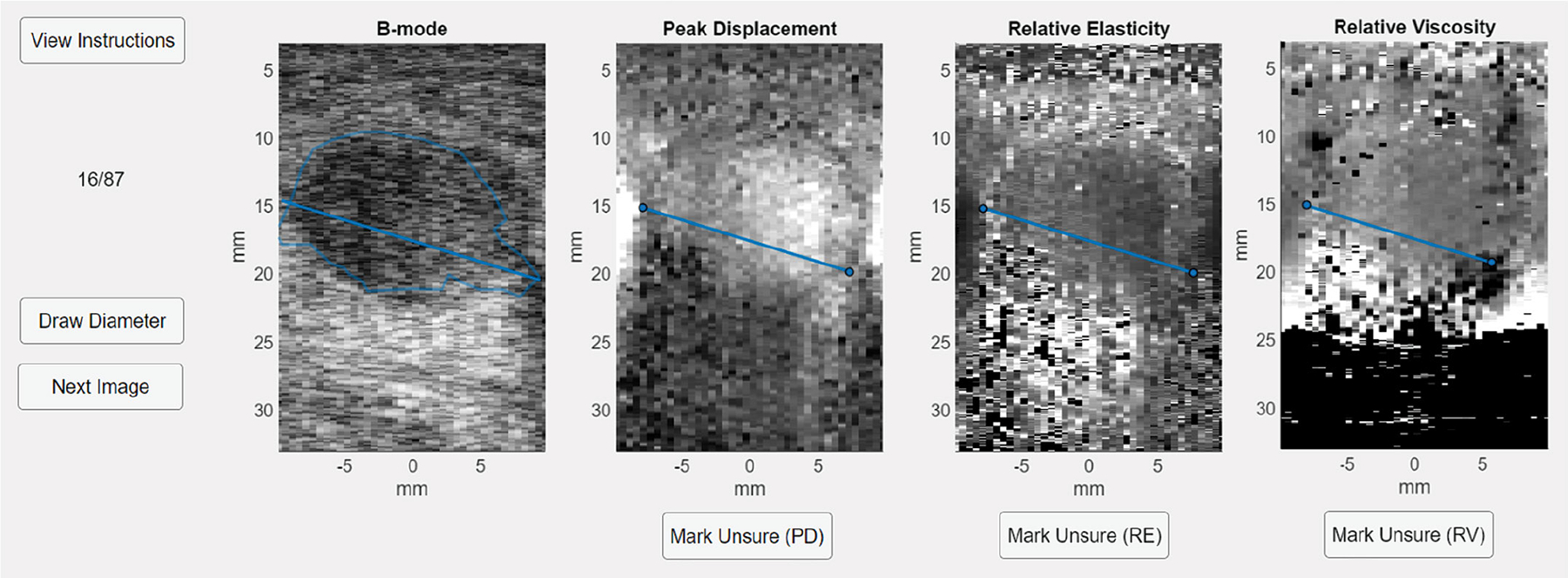
A custom MATLAB app was used for the blinded reader study. Readers viewed the radiologist annotated B-mode, ARFI PD, and VisR RE and RV images and measured the diameters using a line region of interest (ROI).

Figure 1 shows the custom MATLAB app constructed for the reader study. For each lesion view, the radiologist-annotated B-Mode and unannotated PD, RE, and RV images were displayed side-by-side using the same image resolution (2049 axial × 40 lateral data points), size, and gray colormap. This display scheme was chosen to minimize confounding effects from differing image dimensions, resolution, or colormaps in comparing lesion boundaries between the four image types. The 128 lesion examples were shown to each reader in randomized order and without any malignant/benign labeling.

Six research engineer readers participated in the study. The readers were ultrasound engineers who were familiar with B-Mode, PD, and VisR RE and RV images, but were not radiologists or breast imaging specialists. One reader was a trained sonographer. Readers received ~20 minutes of training on how to identify lesion boundaries in PD, RE and RV images, use the app, and measure lesion diameters. None of the images used for training were included in the study dataset. Two methods for lesion diameter measurement were tested by each reader using two slightly different versions of the app. First, they went through the entire dataset using the “manual” measurement method, and then again using the “auto” method. For both methods, readers were instructed to measure the diameter at the largest extent of the lesion, as the most contemporary guidelines suggest [15]. Additionally, readers were instructed to mark any PD, RE, or RV image in which the lesion boundaries could not be visually recognized as “unsure” using the app, and these images were excluded from E/B analysis. This exclusion was intended to both estimate the proportion of low-quality images in the dataset and reduce the impact of low-quality images on the results.

In “manual” mode, readers were asked to determine the largest extent of each lesion based on their visual perception of the radiologist-annotated B-Mode image. They drew the diameter at this location using a line region of interest (ROI) tool, and the line was automatically copied to the corresponding location on the PD, RE, and RV images. The line ROI after being copied was fixed such that it could not be translated or rotated, but the endpoints could be manually adjusted on each image to match the perceived lesion boundaries. Since the B-Mode, PD, RE, and RV data were derived from the same raw data, the corresponding locations in the images represent the same underlying anatomy. In “auto” mode, the radiologist-annotated lesion boundary was used to automatically determine the largest dimension of the lesion on B-Mode and place the diameter line ROI there. The line ROI was copied to the PD, RE, and RV images, and endpoints were adjusted by the reader to match lesion boundaries, as in the manual mode.

After the reader study was completed, E/B ratios were calculated from the data as the ratio of PD, RE, or RV diameter length to B-Mode diameter length. The E/B data was used to construct a binary classifier logistic regression model between malignant and benign. Models were made from each reader’s data using PD-, RE-, and RV-derived E/B individually and in combinations. Lesion characteristics (depth, area, circularity, and PD, RE, and RV degree of anisotropy (DoA)) were also derived from images and correlated with E/B and frequency of being marked unsure. Depth, area, and circularity were calculated from the radiologist B-Mode outline. PD, RE, and RV DoA were calculated from the VisR images as in previous work [25].

## III. RESULTS

**FIGURE 2.**
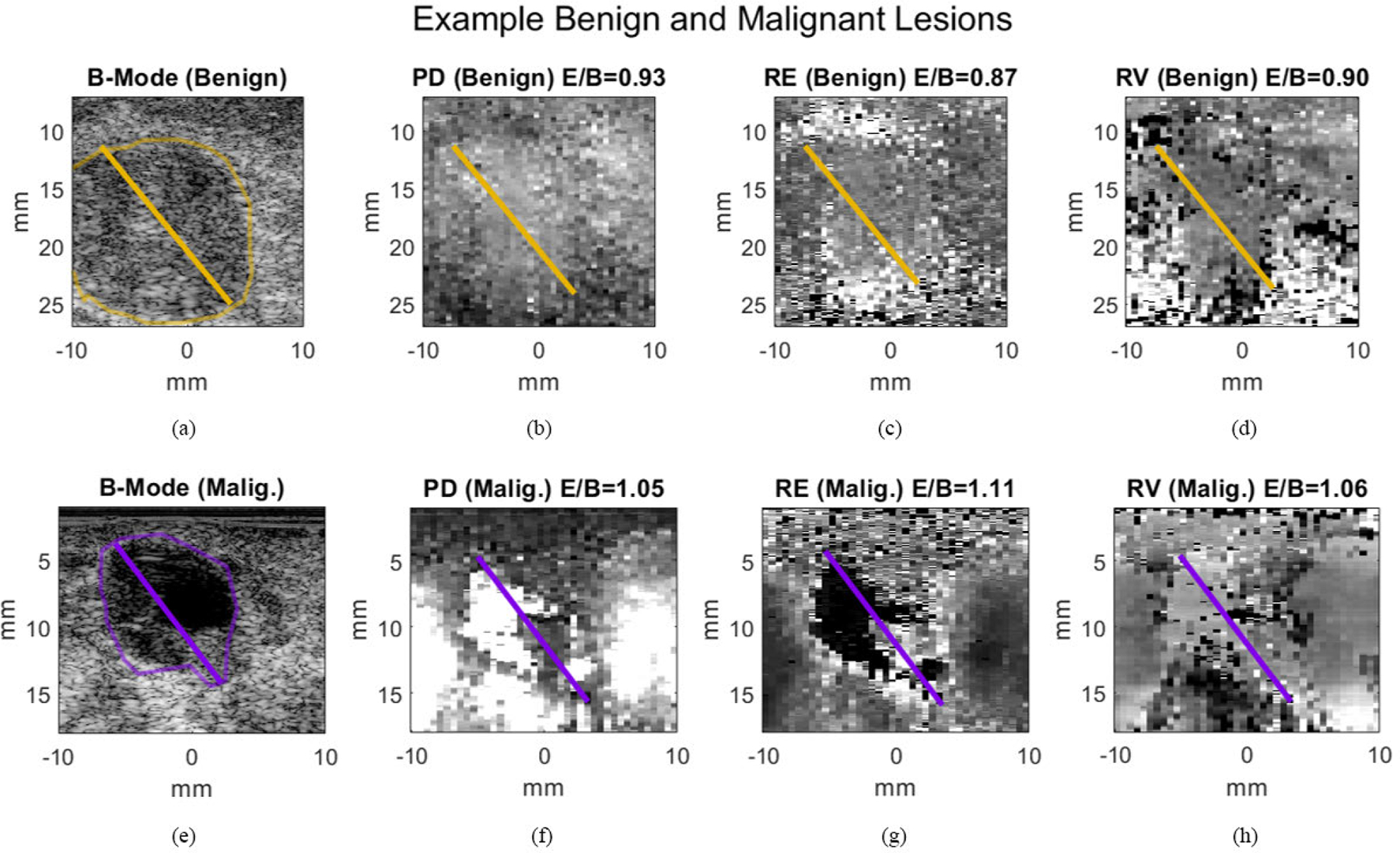
Example images of a benign (a-d) and a malignant (e-h) breast lesions, with diameters manually measured by one of the six readers in B-mode (a,e), PD (b,f), RE (c,g), and RV (d,h). In these examples, all E/B ratios are calculated from PD, RE, and RV images were <1 for the benign lesion and>1 for the malignant lesion.

Figure 2 displays B-Mode, PD, RE, and RV images of sample malignant and benign lesions from the dataset. In the benign lesions, E/B is generally <1 and in the malignant lesions, generally >1, as expected. Additionally, the lesion margins of both have contrasting RE and RV compared to surrounding tissue.

**FIGURE 3.**
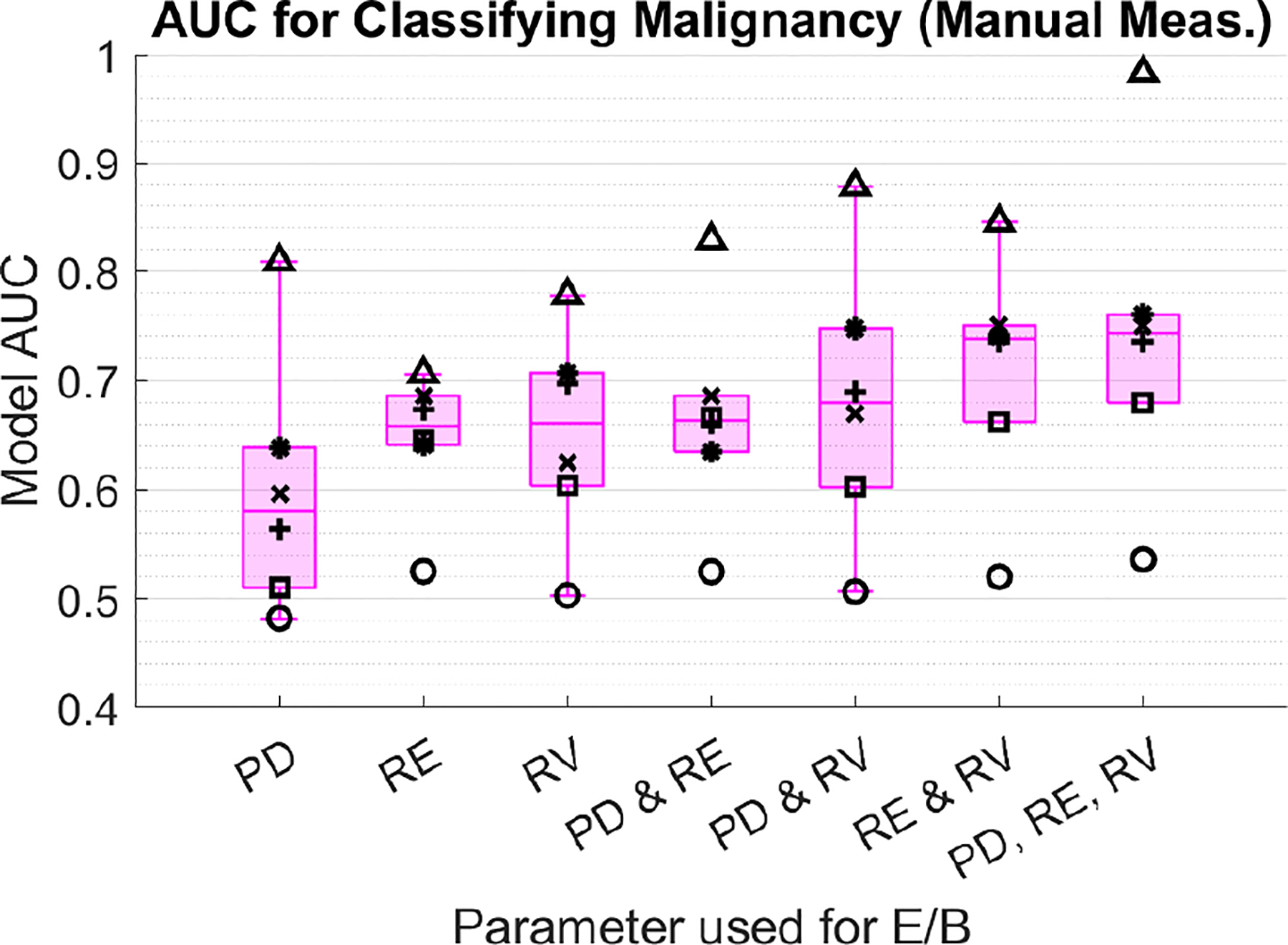
AUC from logistic regression classifier model based on E/B measured from PD, RE, and RV are shown for the manual diameter measurement method. Individual readers are designated by marker shape.

**FIGURE 4.**
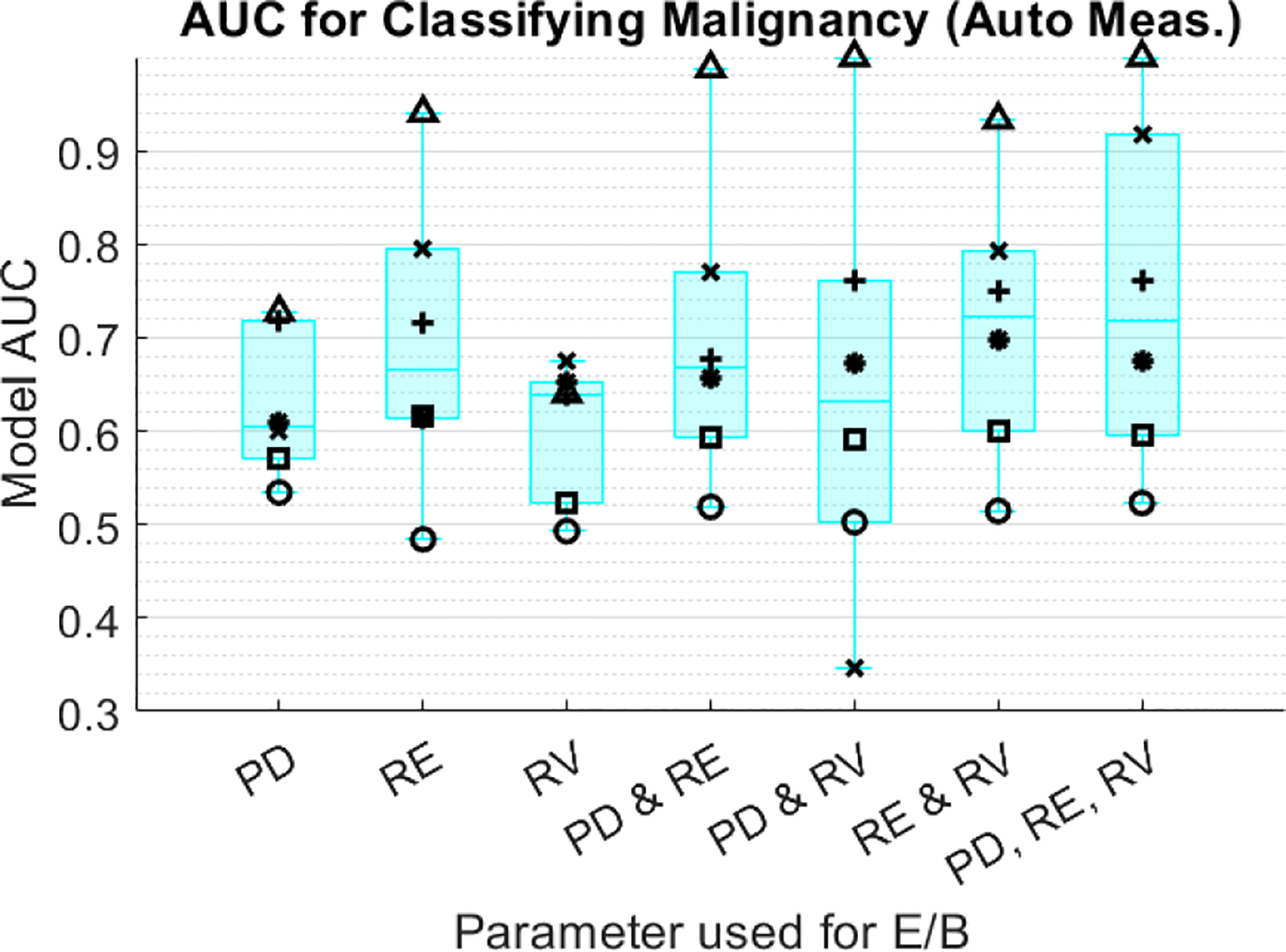
AUC from logistic regression classifier model based on E/B measured from PD, RE, and RV are shown for the automated diameter measurement method. Individual readers are designated by marker shape.

Linear regression model results for classifying malignancy using PD-, RE-, and RV-derived E/B ratio are shown in Figures 3 and 4. Figure 3 shows individual reader AUC for each model using the manual diameter measurement method, and Figure 4 shows equivalent results for the automated measurement method. Model AUC varies substantially between readers but is not significantly different between the manual and auto measurement methods (p>0.05, Wilcoxon Rank Sum). The AUC for RE and RV-derived models is higher than for PD-derived models. Models using E/B from both RE and RV perform the best, with average AUC=0.73 between all readers. Adding PD E/B to the RE and RV model does not further improve AUC. Average number of E/B samples per model across all readers are shown in Tables 1 and 2. Model size is not significantly different (p>0.05, Wilcoxon Rank Sum) between manual and automated methods. Overall, readers had good agreement in the placement of their diameter measurements in both the manual and automated methods, but the length of diameter measured varied between readers for a given image. The best performing readers had significantly different (p<0.05, Wilcoxon Rank Sum) measured E/B between benign and malignant cases in all three image types for both methods.

Correlation between E/B and lesion characteristics is shown in Tables 3 and 4. Reader measured E/B generally had very weak correlation (R<0.19) with lesion characteristics, other than area and RE DoA, which had weak (R = 0.20–0.39) negative correlations.

**FIGURE 5.**
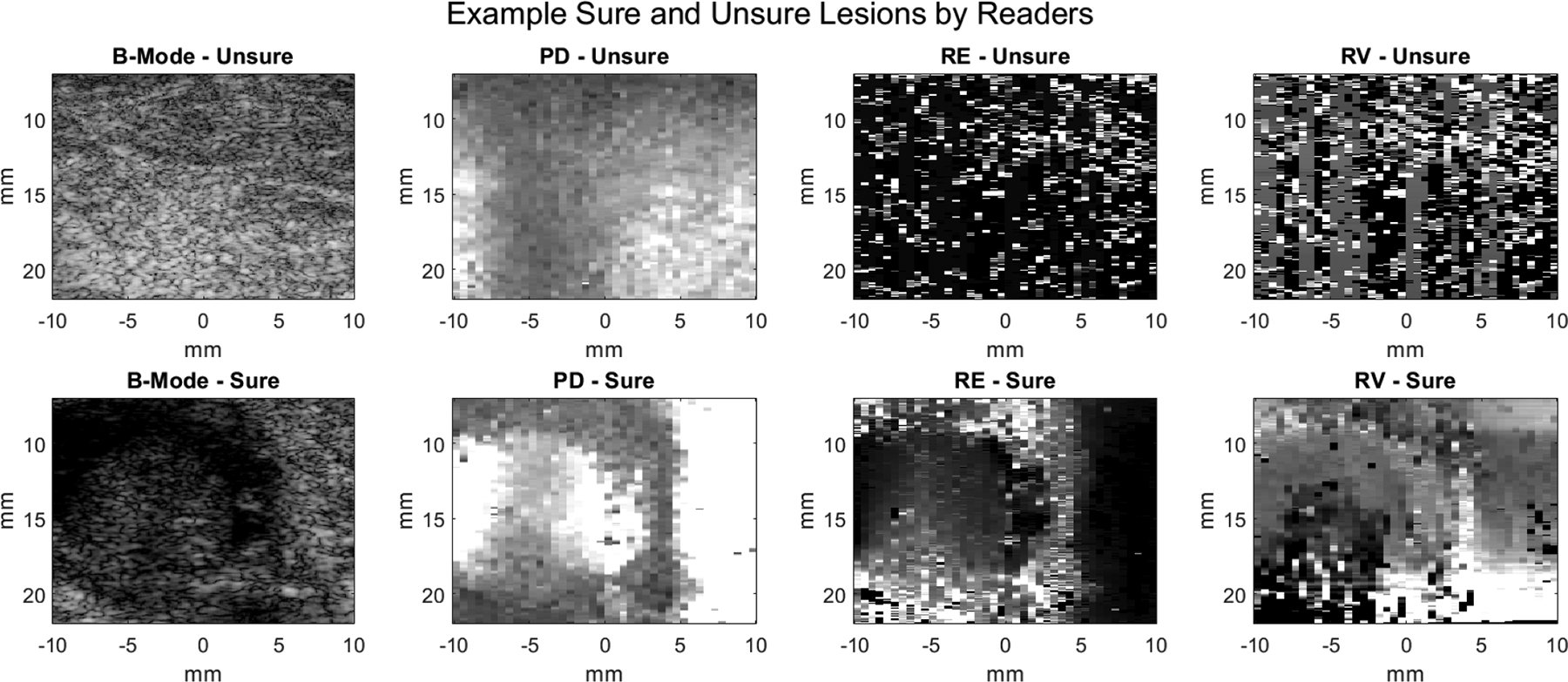
The top row shows PD, RE, and RV images from the lesion most frequently marked unsure by the readers, with corresponding B-Mode. The bottom row shows PD, RE, and RV images with corresponding B-Mode from a lesion that was not marked unsure by any of the readers.

The frequency that a given image was marked unsure by readers can be used as a quality metric. Figure 5 shows the PD, RE, and RV images for the lesion that was marked unsure the most frequently by readers, as well as the corresponding B-Mode. The images notably lack visual contrast between the lesion and background. In comparison, a lesion that was marked unsure zero times by the readers has visually identifiable boundaries in PD, RE, and RV, as well as the corresponding B-Mode.

**FIGURE 6.**
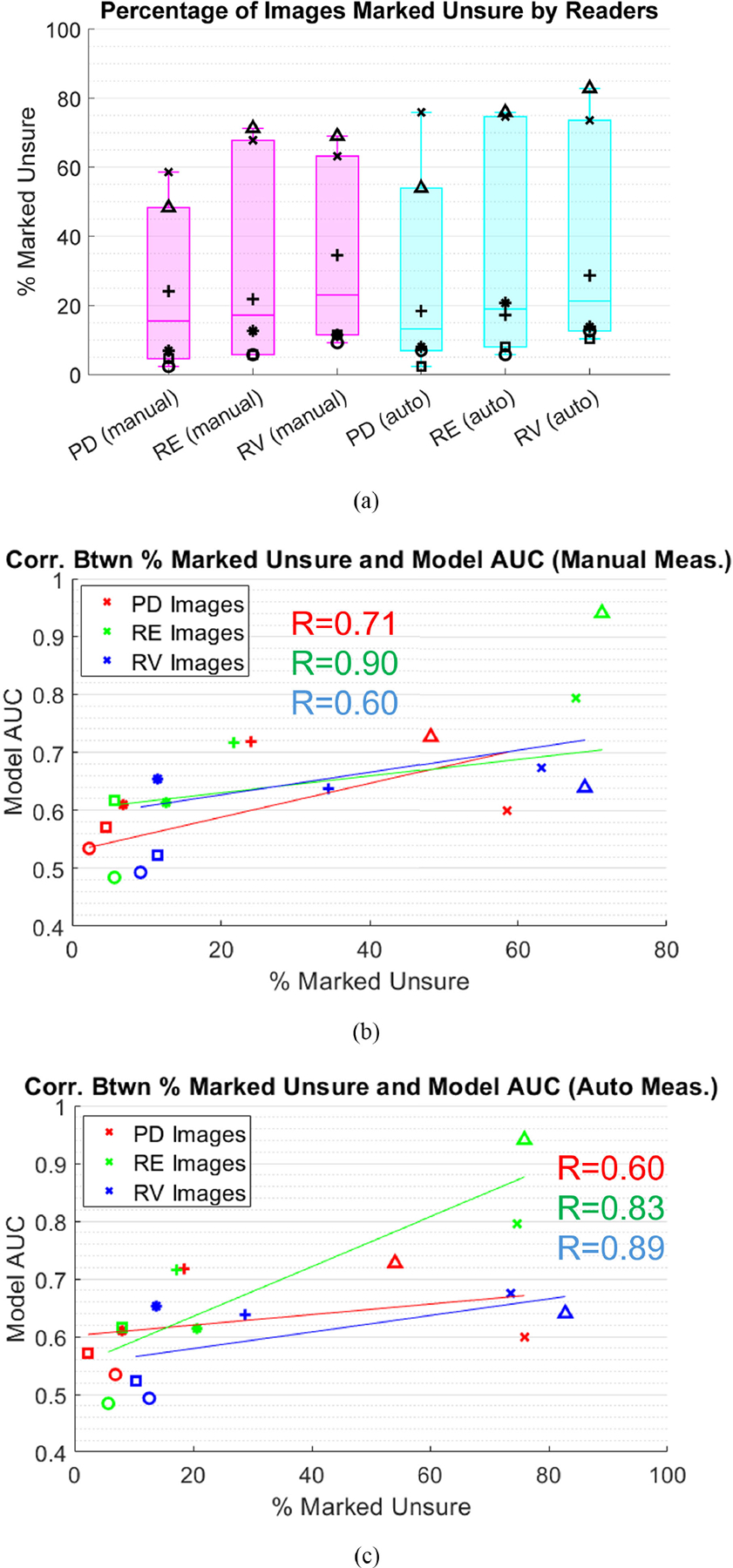
In (a), percentage of images marked unsure by the readers in manual and automated measurement trials are shown. Spearman correlation coefficients between percentage of PD, RE, and RV images marked unsure and E/B model AUC are shown in (b) and (c). Individual readers are designated by marker shape.

Percentage of lesions marked unsure between the manual and auto measurement methods are shown in Figure 6A. Differences between manual and automated, as well as between PD, RE, and RV, are insignificant (p>0.05, ranksum). On average, 15–25% of the images were marked unsure by each reader, but standard deviation between readers is large (~30%). Individual readers were consistent in number marked unsure between PD, RE, and RV and between manual and auto measurement techniques. Correlation between amount of PD, RE, and RV images marked unsure and AUC of PD-, RE-, and RV-derived E/B models is shown in Figures 6B and 6C. Models derived from PD (0.6–0.7) and RV (0.6–0.9) have moderate correlation with number excluded, while models derived from RE (0.8–0.9) have strong correlation.

**FIGURE 7.**
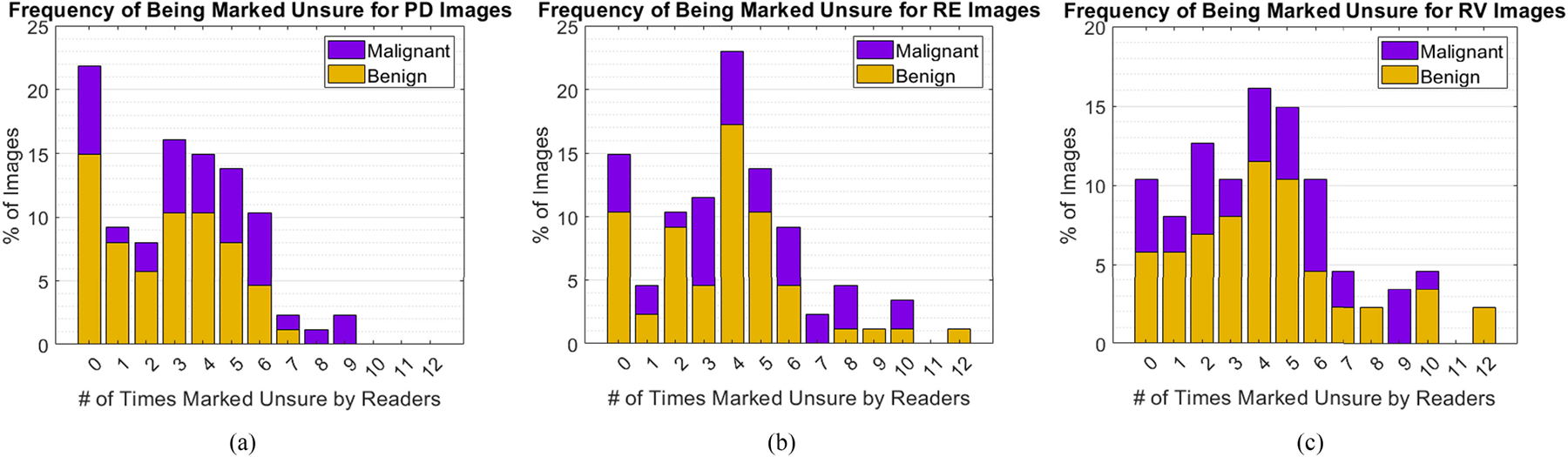
Frequency distributions for number of times each type of image was marked unsure by readers are shown in (a), (b), and (c), with benign images in yellow and malignant in purple.

Proportion of malignant and benign cases compared to frequency of being marked unsure by readers is shown in Figure 7 (a–c). The overall dataset is ~65% benign and ~35% malignant, and this balance is largely maintained even in lesions that were marked unsure by multiple readers. Most lesions were marked unsure fewer than 6 times across all readers and between manual and auto methods. Several were never marked unsure (22% of PD, 15% of RE, and 10% of RV). In RE ~1% of images and in RV ~2% of images were marked unsure every time. Overall, PD images were marked unsure least frequently. Lesion characteristics are also correlated with frequency of being marked unsure in Table 5. Area, circularity, and DoA have negligible (R<0.3) correlation with being marked unsure, but focal depth has mild correlation.

**FIGURE 8.**
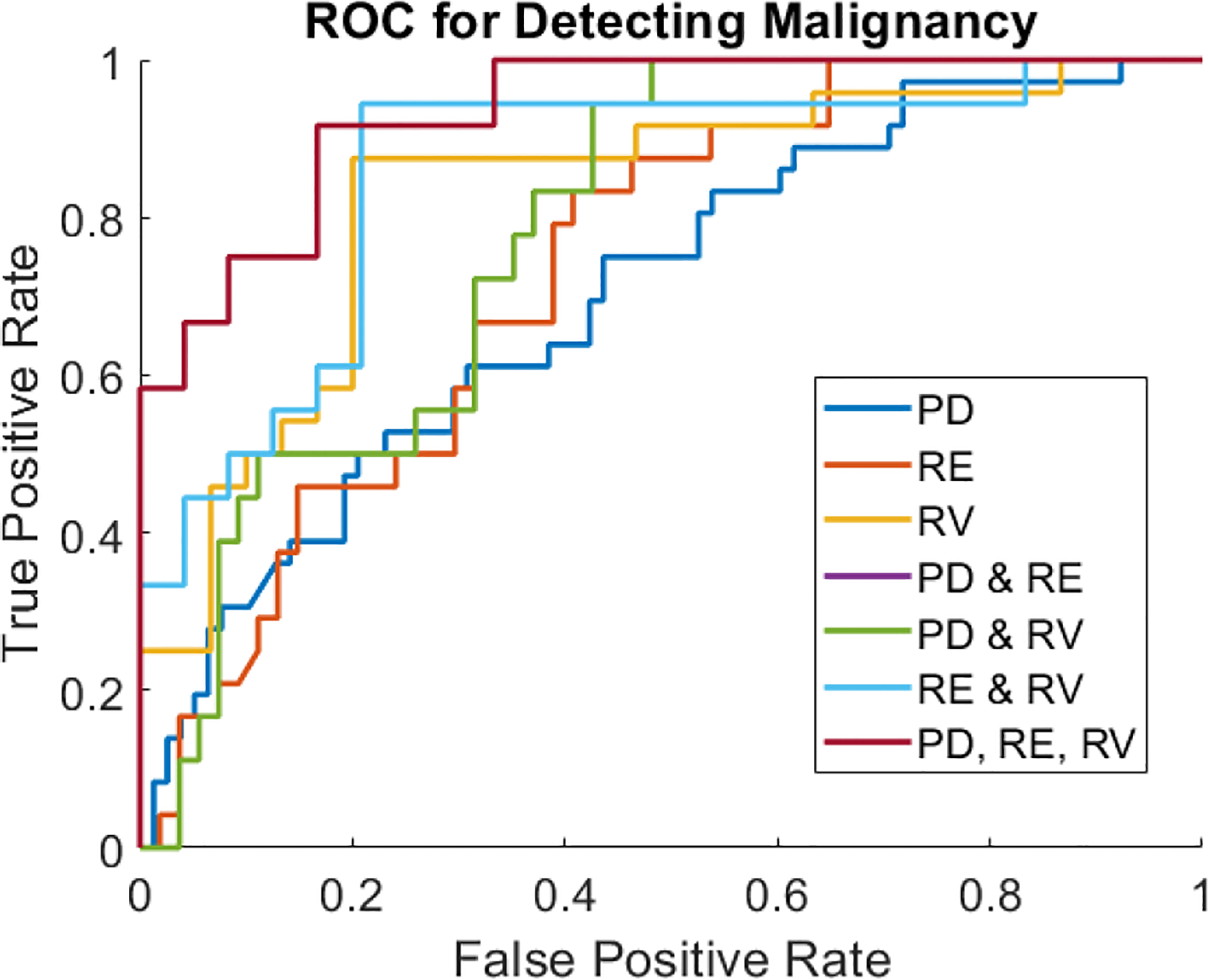
Reciever operating characteristic (ROC) curves are shown for “optimal” models using only images approved (not marked unsure) by all readers.

Based on the correlation we observed between number of images marked unsure and improved model performance, we assumed that images that were never marked unsure by any of the readers (in either the manual or automated diameter studies) were the highest quality. To investigate the performance of VisR-based E/B in an “optimal” diagnostic scenario of best-quality data, we created another set of models combining all reader data using the never marked unsure images. The manual measurement data was used, as there was no significant improvement in performance from using the automated measurement method. ROC curves for the “optimal” models are shown in Figure 8. As shown in Table 6, E/B based on PD, RE, and RV individually had improved AUC over the across-reader median of the initial reader studies. Further, the combined model of PD, RE, and RV achieved the best AUC of 0.93 as well as highest specificity of 1.00. The RE and RV combined model had the best sensitivity of 0.94.

## IV. DISCUSSION

Our expectation that E/B models derived from VisR RE and RV would outperform those from ARFI PD was validated through higher average AUC. In the initial reader study, when all of the reader-marked images were considered, models with RE- and RV-derived E/B combined performed the best and were not improved by adding PD- derived E/B. When only the “optimal” images that were read by all readers were considered, AUC for PD-, RE-, and RV-derived E/B was improved, and the combination of the three parameters had the best AUC. In malignant lesion margins stiffness, viscosity, or both may be increased compared to surrounding tissue. This is supported by RE- and RV-derived E/B being higher on average in malignant lesions. Both increased viscosity and increased stiffness result in lower peak displacement. Because of this, high viscosity with low stiffness and low viscosity with high stiffness could result in similar PD values. This confounding effect could have caused underestimation of lesion size in some malignant PD images, potentially explaining why PD-derived E/B had lower sensitivity and AUC for detecting malignancy. In contrast, RE and RV may provide greater sensitivity by independently detecting stiffness and viscosity. The RE and RV combined model had the best sensitivity, supporting this idea. However, it is unknown why adding PD to this model improved specificity.

Lesion characteristics like depth, circularity, and mechanical anisotropy had negligible correlation with PD-, RE-, or RV-derived E/B. This is encouraging for clinical use as it indicates that any of the evaluated E/B measurement approaches are robust to different imaging focal depths and lesion shapes. It is also important for E/B to be robust to different levels of tissue anisotropy, as breast tissue can vary between negligibly and significantly anisotropic [25], [26]. Lesion area is potentially impactful on the E/B metric, and this effect should be studied further. A potential cause could be the differing surface area to volume ratio in larger lesions affecting the width of the tumor boundary area where mechanical properties are altered with malignancy.

Reader training is an area for improvement, as variation in model AUC and number of images marked unsure between readers was high. Readers were internally consistent in both model AUC and images marked unsure, indicating differing interpretation of the lesion measurement criteria. The automated method for selecting diameter measurement location may not be relevant to improving model performance, as it did not improve average model AUC. Additionally, diameter measurement location on the lesions was consistent between readers and between the manual and automated methods, indicating that readers were capable of judging the largest dimension of a lesion without assistance. The readers differed substantially in their judgement of the endpoints of lesion diameter lines, which means clarification on how to interpret lesion boundaries would be helpful. For example, creating criteria like including or excluding the outermost edges of the lesion margins in the diameter measurement could improve results. The impact of more specific measurement criteria on reader agreement and model accuracy could be explored in future work. In clinical practice, using multiple reviewers for evaluating E/B from images may help mitigate variability. If an image is highly uncertain (multiple readers are unable to discern the lesion boundaries), reporting guidelines could involve excluding it from E/B calculations or flagging it as low confidence.

Since the number of images marked unsure was moderately to strongly correlated with E/B model AUC in the initial reader study, E/B likely performs better as a diagnostic metric when higher quality lesion images are used. This idea is supported by the improved performance of the “optimal” models. However, this result could be affected by model overfitting on smaller datasets, so a larger study is needed to confirm. Still, the best performing initial reader models were not significantly (p>0.05, Wilcoxon Rank Sum) smaller than the worst performing models. The correlation between model size and AUC was strongest in RE, which could mean that RE image quality is the most impactful in building an accurate diagnostic model with E/B. Overall, PD images were marked unsure the least frequently, so they were likely the highest quality, but also had the least impact on model performance. Average image quality being higher in PD than RE and RV may be due to cases where tissue displacement did not fit the VisR mass-spring-damper model well. This can occur from noisy displacement tracking due to signal attenuation and abberation, motion during data acquisition, or from the tissue exhibiting nonlinear viscoelastic behavior. Lesion area, circularity, and anisotropy were not correlated with being marked unsure by readers, but focal depth was. This could indicate that deeper lesions are more poorly depicted, perhaps due to ARFI excitation and tracking beam attenuation that reduce CNR for PD, RE, and RV images.

The average AUC and sensitivity of the readers’ models were lower than in the unblinded preliminary study [23]. This may be due to lack of reader experience with interpreting B-Mode, PD, and VisR breast lesion images. The lateral sampling of the PD, RE, and RV images was limited to keep image acquisition time low, which may have resulted in many low-quality images. Further studies with VisR could test a higher lateral sampling rate, but this must be balanced to stay within feasibility for clinical imaging. The factors impacting VisR image quality and the effect of image quality on diagnostic value of E/B should be a focus of future work. Finally, a larger dataset would strengthen the conclusions of this work, as the present study was limited by clinical data collection at a single medical center. Class balance between malignant and benign was adequate, but multi-parametric logistic regression models are most statistically robust with >100 samples.

## V. CONCLUSION

We assessed the diagnostic benefit of calculating the E/B metric for breast lesions using three on-axis, ARFI-based viscoelastic parameters: peak displacement, relative elasticity, and relative viscosity. In a blinded study of six readers, *in vivo* breast lesion diameters were measured using both a manual and partially automated method. In logistic regression binary classifier models for malignancy, E/B derived from RE and RV had higher AUC than E/B derived from PD. Stiffness and viscosity are biomarkers for breast cancer separately measured by RE and RV, whereas PD is a stiffness estimate that is confounded by viscosity. Combining E/B from PD, RE, and RV and using the highest-quality VisR images further enhanced accuracy and achieved an AUC of 0.93 for discriminating malignant lesions. Diagnostic accuracy was similar for the manual and automated E/B measurement methods, supporting the established methodology for calculating E/B by measuring in the reader-determined largest lesion dimension. We found low correlation between E/B and lesion characteristics like shape, depth, and tissue anisotropy, supporting the use of VisR-based E/B as a robust clinical metric. Finally, we found a strong correlation between low-quality images being excluded and improved model AUC, but further analysis with a larger dataset would strengthen this conclusion. Future work exploring the use of VisR for breast cancer diagnosis should focus on improving image quality, image quantity, and reader training. Overall, this study shows increased accuracy in breast cancer diagnosis when VisR viscosity and stiffness images are both used to calculate E/B.

## Figures and Tables

**TABLE 1.** Logistic regression model Avg. # of E/B samples (Manual Meas).

E/B Parameter	Average	Std. dev.
Peak Displacement	N=64	20
Relative Elasticity	N=65	25
Relative Viscosity	N=56	22
PD and RE	N=49	24
PD and RV	N=51	25
RE and RV	N=61	26
PD, RE, and RV	N=47	26

Average number of E/B samples per model and standard deviation from the manual diameter measurement method are shown.

**TABLE 2.** Logistic regression model Avg. # of E/B Samples (Auto Meas).

E/B Parameter	Average	Std. dev.
Peak Displacement	N=74	26
Relative Elasticity	N=68	27
Relative Viscosity	N=67	27
PD and RE	N=64	29
PD and RV	N=62	28
RE and RV	N=64	26
PD, RE, and RV	N=59	26

Average number of E/B samples per model and standard deviation from the automated diameter measurement method are shown.

**TABLE 3.** Avg. Corr. Coeff. between reader E/B and lesion properties (Manual Meas).

Lesion Property	PD E/B	RE E/B	RV E/B
Focal Depth	−0.03	−0.05	0.01
Area	−0.24	−0.23	−0.21
Circularity	0.09	0.15	0.11
PD DoA	−0.05	0.01	0.10
RE DoA	0.11	0.22	0.07
RV DoA	−0.17	−0.14	−0.03

Pearson correlation coefficient was calculated between E/B and lesion properties and averaged across readers. This table shows correlation coefficients for the manual measurement method.

**TABLE 4.** Avg. Corr. Coeff. between reader E/B and lesion properties (Auto Meas).

Lesion Property	PD E/B	RE E/B	RV E/B
Focal Depth	0.04	0.06	0.23
Area	−0.24	−0.29	−0.25
Circularity	0.05	0.14	0.13
PD DoA	−0.04	0.00	−0.01
RE DoA	0.22	0.29	0.16
RV DoA	−0.17	−0.20	−0.03

Pearson correlation coefficient was calculated between E/B and lesion properties and averaged across readers. This table shows correlation coefficients for the automated measurement method.

**TABLE 5.** Corr. Coeff. between times marked unsure by all readers and lesion properties.

Lesion Property	PD Images	RE Images	RV Images
Focal Depth	0.32	0.42	0.26
Area	−0.15	0.03	0.12
Circularity	0.20	0.19	0.10
PD DoA	−0.08	−0.22	−0.11
RE DoA	0.37	0.21	0.27
RV DoA	0.21	0.09	0.18

Spearman correlation coefficients between lesion characteristics and number of times the image was marked unsure by readers are shown.

**TABLE 6.** “Optimal” images model performance.

E/B Parameter	AUC	Sens.	Spec.	Model Size
PD	0.70	0.28	0.94	N=114
RE	0.74	0.46	0.85	N=78
RV	0.83	0.88	0.80	N=54
PD and RE	0.78	0.39	0.93	N=72
PD and RV	0.87	0.89	0.80	N=48
RE and RV	0.86	0.94	0.79	N=42
PD, RE, RV	0.93	0.58	1.0	N=36

The performance of logistic regression models using only images approved (not marked unsure) by all readers is shown by AUC, sensitivity, and specificity. Models were tested for PD-, RE-, and RV-derived E/B, and combinations of the three. The number of samples in each model is shown in the bottom row.
